# From Cabinet to Catalogue: The Palaearctic bee (Hymenoptera, Apoidea, Anthophila) collection of the Museu Nacional de História Natural e da Ciência, Universidade de Lisboa, Portugal 

**DOI:** 10.3897/BDJ.14.e188597

**Published:** 2026-06-25

**Authors:** Martim Baptista, Paulo de Sousa, Roberto A. Keller

**Affiliations:** 1 Museu Nacional de Historia Natural e da Ciência, Universidade de Lisboa, Lisbon, Portugal Museu Nacional de Historia Natural e da Ciência, Universidade de Lisboa Lisbon Portugal; 2 Centre for Ecology, Evolution and Environmental Changes & CHANGE - Global Change and Sustainability Institute, Universidade de Lisboa, Lisbon, Portugal Centre for Ecology, Evolution and Environmental Changes & CHANGE - Global Change and Sustainability Institute, Universidade de Lisboa Lisbon Portugal; 3 General Zoology, Institute of Biology, Martin-Luther-Universität Halle-Wittenberg, Halle, Germany General Zoology, Institute of Biology, Martin-Luther-Universität Halle-Wittenberg Halle Germany

**Keywords:** Andrenidae, Apidae, Colletidae, Halictidae, Megachilidae, Melittidae, pollinators, museum collections, new record, type material

## Abstract

**Background:**

The Mediterranean Basin, characterised by temperate rainy winters and hot dry summers, represents a notable hotspot for bee species richness. However, research on the bee fauna across this region remains uneven, with significant knowledge gaps in countries like Portugal. As human-driven climate change continues to cause a dramatic decline on insect species, museum records become indispensable for assessing changes in species distributions over time and to preserve material evidence about species for future generations.

**New information:**

We provide data on 5,295 bee records with Palaearctic distribution, collected from the 1920s to the year 2025. The collection, hosted in the Museu Nacional de História Natural e da Ciência (MNHNC), Universidade de Lisboa (UL), encompasses 425 species and 49 genera. The majority of the records originate from Portugal, including a newly-documented species for the country, *Nomada chrysopyga* Morawitz, 1871, bringing the total number of bee species in mainland Portugal to 737. Type material held in the collection is documented and photographed here, alongside a record of bee types destroyed in the 1978 fire.

## Introduction

Bees (Hymenoptera, Apoidea, Anthophila) perform a crucial role as pollinators in all biomes where they occur (Ollerton 2017), as females have evolved specialised hairs and body structures used to collect pollen, that they use mainly for larval provisioning (Thorpe 1979, Michener 2007). They are vital to both wild plants and crop production worldwide (Potts et al. 2016, Klein et al. 2018), due to promoting plant growth and reproduction while fostering genetic diversity within floral populations (Loveless and Hamrick 1984, Jha and Dick 2010).

Bee population declines have become a matter of growing environmental concern (Decourtye et al. 2019, Lima et al. 2022, Panziera et al. 2022). Pesticides and, to a certain degree, habitat loss are amongst the most pervasive threats to wild bees (Goulson et al. 2015, Ollerton 2017, Guzman et al. 2024), while competition with honey bees and disease spillover from domestic to wild populations also contribute to the instability of native populations (Fürst et al. 2014, Mallinger et al. 2017, Alaux et al. 2019). Population decline in temperate regions has led to reduced levels of genetic diversity (Kelemen and Rehan 2021), which leaves these populations more vulnerable to environmental change and decreases their chances of survival in the long run. The cumulative effect of these threats makes studying and conserving these insects increasingly pressing.

The Palaearctic Region includes important hotspots of bee diversity, notably the Middle East and the Mediterranean Basin (Michener 2007, Orr et al. 2023). The Mediterranean's abundant solar exposure, dry summers and rainy mild winters and diminished seasonal variation lead to high flora diversity which favours the high number of bee species in the region (Cowling et al. 1996, Petanidou and Ellis 1996, Orr et al. 2023). Bees also have a preference for open environments with low vegetation (Nery et al. 2018, Wu et al. 2018), which includes the dry shrublands common to the Mediterranean Basin. Moreover, the frequent fires in the region help clear vegetation away and leave the ground bare, creating suitable nesting sites for various bee genera (Potts et al. 2003, Potts et al. 2005, Antoine and Forrest 2021, Brokaw et al. 2023). Within the Mediterranean area, the Iberian Peninsula has an abundance of endemic species (Ascher and Pickering 2023, Michez et al. 2026), probably due its isolation from other regions in Europe and Africa, multiple mountain chains and local abiotic conditions (Blondel et al. 2010). Furthermore, it has served as a climatic refugia for several species during the Pleistocene glaciations (Gómez and Lunt 2007), including for both solitary and social bee species (Chávez-Galarza et al. 2015, Silva et al. 2020, Sousa et al. 2023).

Before the present study, the Portuguese bee fauna included 736 bee species on the mainland (Reverté et al. 2023, Gaspar et al. 2025, Le Divelec and Michez 2025, Ghisbain et al. 2025), of which more than 40 are Iberian endemics (Wood et al. 2020, Cross 2023, Wood 2023, Le Divelec and Michez 2025). A total of 54 genera can be found in the country (Baldock et al. 2018, Ghisbain et al. 2025), the most speciose being *Andrena* Fabricius, 1775, as is common across Holarctic countries (Wood 2023), with 128 species. Anthophorine and Panurgine bees are also significantly diverse, a trend common in xeric Mediterranean countries (Baldock et al. 2018, Wood et al. 2020, Lhomme et al. 2020, Reverté et al. 2023). New records for Portugal and new species have recently been uncovered at a rapid rate as large previously understudied parts of the country have been investigated (Wood and Cross 2017, Wood et al. 2020, Soares et al. 2022, Cross 2023, Gaspar et al. 2023, Gaspar et al. 2025). However, despite advances being been made in recent years to sample these lesser-studied regions of the country (Gaspar et al. 2023), information on past and present records remains incomplete. To address these gaps, the ARCADE project is undertaking the revision and digitisation of the main pollinator collections held in the country. A dataset on the bees of the Museu de Ciência da Universidade de Coimbra (MCUC), has already been published (Gaspar et al. 2025), with the present work continuing this effort.

Natural history museums are the backbone of biodiversity studies and are indispensable sources for taxonomists, evolutionary biologists, ecologists and conservation biologists (Wheeler 2023). They provide primary data on species taxonomy and distribution, support accurate species identification and preserve critical information on morphology and evolutionary history (Colvin 2014, ESA 2021, Wheeler 2023). In addition, these collections offer historical baselines that enable researchers to document changes in insect communities in response to environmental change (Colvin 2014) and to trace spatial and temporal patterns in genotypic and phenotypic variation (Jensen et al. 2022, Mullin et al. 2022). The growing recognition of pollinators' importance makes it all the more pressing to digitise these collections and make their holdings — type specimens in particular — openly accessible (Corona 2023, Meunier et al. 2023, Gaspar et al. 2025).

## General description

### Purpose

The aim of this work is to promote the study of bee specimens housed at the Museu Nacional de História Natural e da Ciência (MNHNC, Universidade de Lisboa), Lisbon, Portugal, by making data on their diversity freely and remotely accessible through the Global Biodiversity Information Facility (GBIF). The dataset includes information on specimen counts, levels of taxonomic identification and the researchers responsible for each identification. Data on sampling locality and collection date support analyses of the distribution and seasonality of bee taxa occurring in Portuguese territory. In addition, we provide images of the bee type specimens preserved at MNHNC.

## Project description

### Title

Palaearctic bee collection of the Museu Nacional de História Natural e da Ciência, Universidade de Lisboa, Portugal

### Funding

This study was supported by the Portuguese Research Infrastructure of Scientific Collections, co-financed by FCT (Fundação para a Ciência e a Tecnologia) and FEDER (Fundo Europeu de Desenvolvimento Regional), and by PORBIOTA – E-Infraestrutura Portuguesa de Informação e Investigação em Biodiversidade (ref. 22127). The article is part of the ARCADE project, which has received funding from the European Union's Horizon Europe Research and Innovation Programme under Grant Agreement No. 101081903. M.B. was supported by ULisboa scholarship 10/BI/2022. P.S. was supported by ULisboa scholarship 06/BI/2021. R.A.K. was supported by FCT https://doi.org/10.54499/DL57/2016/CP1434/CT0001. Additional financing was provided by the ce3c research unit through FCT funding https://doi.org/10.54499/UID/00329/2025.

## Sampling methods

### Sampling description

The Palaearctic bee specimens housed at the MNHNC were collected between 1926 and 2025, primarily from 580 localities in Portugal, with additional sporadic samples originating from 55 other Palaearctic localities. Notable contributions to the collection include those by David W. Baldock (2,105 records), Mike and Elizabeth Howe (1,235 records) and the Mendoça family (428 records). Additionally, material from the Estações da Biodiversidade (EBIO) Network in Portugal (167 records), which surveyed across biological monitoring stations throughout the country, significantly enhanced the collection's diversity, particularly from central and northern Portugal. The integration of the now-defunct Instituto de Investigação Científica Tropical (IICT) into the University of Lisbon in 2015, followed by the transfer of its insect collection to the MNHNC, further enriched the Palaearctic bee collection (104 records), most of which date to the first half of the 20^th^ century.

### Quality control

Hymenopterists on whose identifications we depended are: David W. Baldock (families Apidae, Andrenidae, Colletidae, Halictidae, Megachilidae and Melittidae), Thomas J. Wood (families Apidae, Andrenidae, Colletidae, Halictidae, Megachilidae and Melittidae), Martim Baptista (families Apidae, Andrenidae, Colletidae, Halictidae, Megachilidae and Melittidae), Gérard Le Goff (family Megachilidae and genus *Anthophora*), Erwin Scheuchl (genus *Andrena*), Andreas W. Ebmer (family Halictidae), Bogdan Tomozii (genus *Andrena* and *Hylaeus*), Zsolt Józan (mainly families Halictidae and Andrenidae), Maximilian Schwarz (families Apidae and Halictidae), Francisco J. Ortiz-Sanchez (family Megachilidae, subfamily Anthophorinae and genus *Hylaeus*), Michael Kuhlmann (genus *Colletes*), Leopoldo Castro (genus *Bombus*), Stephan Risch (genus *Eucera*), Ian Cross (families Apidae and Megachilidae), Andreia Penado (genus *Apis*, *Bombus* and *Andrena*), Félix Torres (family Megachilidae), Alain Pauly (family Halictidae), Jan Smit (genus *Nomada*), Sébastien Patiny (genus *Panurgus*), Andreas Müller (genus *Hoplitis* and *Osmia*), Holger H. Dathe (genus *Hylaeus*), Jakub Straka (genus *Sphecodes*), Petr Bogusch (genus *Epeolus* and *Sphecodes*), Christophe J. Praz (genus *Megachile*), Denis Michez (genus *Dasypoda*), Paulo de Sousa (genus *Dasypoda* and *Osmia*), Donald B. Baker (genus *Ammobates*, *Hoplitis* and *Rhodanthidium*), Martin Jenner (genus *Anthophora*), Stuart P.M. Roberts (genus *Ceratina*), Christian Schmid-Egger (genus *Rhodanthidium*), Miguel Azevedo (genus *Megachile*), Paul H. Williams (genus *Bombus*) and Albano Soares (genus *Nomada*).

Nomenclature used for the material in the collection is consistent with the most current European bee checklist (Ghisbain et al. 2025).

### Step description

The digitisation and cataloguing of the collection began in 2013, representing the first concerted effort to create a comprehensive digital database. Prior to this, only a limited number of specimens had their information recorded in paper catalogues. A major update was implemented in 2021, when the structure of the database was redesigned to comply with Darwin Core standards, resulting in the present version.

Between 2021 and 2025, the entire collection was catalogued and georeferenced. Catalogue work included the transcription of specimen label data into the digital database and the assignment of a unique catalogue number to each specimen through the addition of a standardised catalogue label. Georeferencing was performed using the GEOLocate platform (https://www.geo-locate.org/), ensuring the generation of consistent and repeatable geographic coordinates. Locality data are presented in the form of decimal degrees using the WGS84 map datum.

During this period, specimen identifications were critically revised across most genera and previously unidentified material was examined and assigned to the lowest feasible taxonomic rank. Full species-level identification was not achieved for a subset of specimens of *Andrena* Fabricius, 1775 and for all specimens of *Lasioglossum* Curtis, 1833, owing to taxonomic complexity and the need for further study. These specimens remain identified at higher taxonomic levels and their records will be updated in the database as identifications are refined in the future.

Images of the types and of the new record were taken with a Leica DMC4500 camera attached to a Leica Z6 APO microscope on a motorised focus stand, to produce raw photo stacks processed to single montage images with Leica LAS X.

## Geographic coverage

### Description

**Figure 1.**
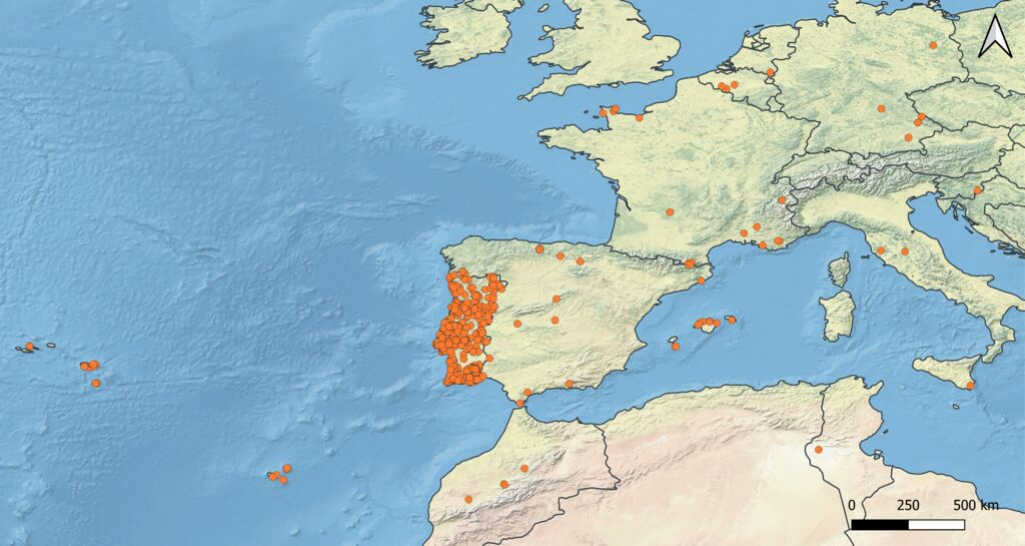
Distribution of Palaearctic bee specimens deposited in the MNHNC insect collection. Specimens with spatial information limited to the level of country of origin are not shown on the map.

The Palaearctic bee specimens originate primarily from Portugal, although there are a few records from eleven other southern and central European and northern African countries (Fig. 1).

### Coordinates

 and Latitude from 31.624813 to 52.515151 Latitude; and Longitude from -28.186176 to 27.527837 Longitude.

## Taxonomic coverage

### Taxa included

**Table:** 

Rank	Scientific Name	Common Name
family	Andrenidae	Mining Bees
family	Apidae	Bumblebees, Honey bees, Carpenter, Cloak and Dagger, Digger, Nomad and Longhorn Bees
family	Colletidae	Plasterer and Yellow-faced bees
family	Halictidae	Halictid Bees
family	Megachilidae	Carder, Leafcutter, Mason, and Resin Bees
family	Melittidae	Melittid Bees

## Temporal coverage

**Data range:** 1926-8-05 – 2025-6-25.

## Collection data

### Collection name

Palaearctic bee collection of the Museu Nacional de História Natural e da Ciência, Universidade de Lisboa, Portugal

### Collection identifier

Each specimen in the collection is assigned a unique identifier, which consists of the institutionCode (MNHNC or INIAV), followed by the collectionCode for the insect collection at each institution (ENT) and a seven or six-digit number (depending on the institution) that ensures the precise identification of each specimen (e.g. MNHNCENT0062868).

### Specimen preservation method

All specimens in the collection are dried and pinned.

## Usage licence

### Usage licence

Creative Commons Public Domain Waiver (CC-Zero)

## Data resources

### Data package title

Palaearctic bee (Hymenoptera, Apoidea, Anthophila) collection the Museu Nacional de História Natural e da Ciência (MNHNC), Lisbon, Portugal

### Alternative identifiers

https://www.gbif.org/dataset/e4bf59a8-2cc6-4cbc-99bf-28d9b4241b91; https://ipt.gbif.pt/ipt/resource?r=palearctic-bees-mnhnc

### Number of data sets

1

### Data set 1.

#### Data set name

Palaearctic bee (Hymenoptera, Apoidea, Anthophila) collection of the Museu Nacional de História Natural e da Ciência (MNHNC), Lisbon, Portugal

#### Data format

Darwin Core Archive format

#### Character set

UTF-8

#### Download URL

https://ipt.gbif.pt/ipt/resource?r=palearctic-bees-mnhnc

#### Data format version

Version 1.7

#### Description

The dataset submitted to GBIF is structured as an occurrence dataset with 5,295 entries (Baptista et al. 2026). The data in this sampling event resource have been published as a Darwin Core Archive (DwC-A), a standardised format common for sharing specimen record datasets.

The 5,295 records include 5,301 specimens, as some pins bear more than one individual and each pin constitutes a single record regardless of the number of specimens it carries. Of these, 5,292 records belong to the MNHNC core collection (catalogue number formated as MNHNCENT#, with MNHNC being institutionCode, ENT being collectionCode and # being a seven digit number) and three are from the former collection of the *Laboratório de Biologia Florestal da Direcção Geral dos Serviços Florestais e Aquícolas*, now part of the Instituto Nacional de Investigação Agrária e Veterinária, I.P. (INIAV) collection (catalogue number formatted as INIAVENT#, with INIAV being institutionCode, ENT being collectionCode and # being a six digit number), all housed in the Museum. A total of 4,830 records are identified to species or subspecies level and 5,267 are georeferenced.

**Data set 1.** 

Column label	Column description
catalogNumber	Unique identifier assigned by the institution to the record within the collection.
occurrenceID	Unique identifier for the record.
eventDate	Date the record was collected.
day	Day the record was collected.
month	Month the record was collected.
year	Year the record was collected.
eventTime	Time at which the record was collected.
verbatimEventDate	The original textual description of the date of collection.
higherGeographyID	Biogeographic region in which the record was collected.
continent	Continent in which the record was collected.
country	Country in which the record was collected.
stateProvince	The name of the next smaller administrative region than country where the record was collected.
island	The name of the island in which the record was collected.
municipality	Municipality in which the record was collected.
locality	Most specific geographic information about the place where the record was collected.
verbatimLocality	The original textual description of the geographic information.
decimalLatitude	Approximate centre point decimal latitude where the record was collected.
decimalLongitude	Approximate centre point decimal longitude where the record was collected.
geodeticDatum	The ellipsoid, geodetic datum or spatial reference system (SRS), upon which the geographic coordinates given in decimalLatitude and decimalLongitude are based.
coordinateUncertaintyInMetres	The horizontal distance (in metres) from the given decimalLatitude and decimalLongitude describing the smallest circle containing the sampling location.
georeferencedBy	Name of the person who georeferenced the record.
georeferencedDate	Date when the specimen was georeferenced.
georeferenceRemarks	Additional information about the georeferencing of the specimen.
verbatimLatitude	The verbatim original latitude where the record was collected.
verbatimLongitude	The verbatim original longitude where the record was collected.
minimumElevationInMetres	The lower limit of the range of elevation (altitude, above sea level), in metres, where the record was collected.
maximumElevationInMetres	The upper limit of the range of elevation (altitude, above sea level), in metres, where the record was collected.
verbatimElevation	The original description of elevation (altitude, above sea level), in metres, where the record was collected.
sex	Biological sex of the record.
lifeStage	Life stage of the record.
caste	Caste of the record.
habitat	Habitat where the record was collected.
fieldNumber	An identifier given to the sampling event.
fieldNotes	Additional information recorded during sampling.
associatedTaxa	Taxa associated with the record (e.g. host plants, parasites) at the time of sampling.
samplingProtocol	Method used for sampling.
recordedBy	Name of the person who collected the specimen.
recordedByID	Unique identifier of the person who collected the specimen.
scientificName	Complete scientific name including author and year.
kingdom	Kingdom name of the record.
phylum	Phylum name of the record.
class	Class name of the record.
order	Order name of the record.
suborder	Suborder name of the record.
superfamily	Superfamily name of the record.
family	Family name of the record.
subfamily	Subfamily name of the record.
tribe	Tribe name of the record.
genus	Genus name of the record.
subgenus	Subgenus name of the record.
specificEpithet	Specific epithet of the record.
infraspecificEpithet	Intraspecific epithet of the record.
taxonRank	Lowest taxonomic rank of the record.
scientificNameAuthorship	Name of the author of the lowest taxon rank included in the record.
identifiedBy	Name of the person who assigned a taxon the record.
identifiedByID	Unique identifier of the person who determined the taxon.
dateIdentified	Date of the last identification of the record.
identificationRemarks	Additional information about the identification.
typeStatus	A nomenclatural type applied to the record.
previousIdentifications	Previous identifications assigned to the record.
verbatimIdentification	Identifications as they appear on the record's original labels.
occurrenceStatus	Confirmation of the presence or absence of the record in the collection.
disposition	The current state of the record with respect to a collection.
basisOfRecord	Nature of the data record.
preparations	Preparation or preservation method of the record.
preparationBy	Name of the person who prepared the record.
preparationDate	Date of the preparation of the record.
individualCount	Number of specimens represented by the record.
cataloguedBy	Name of the person who catalogued the information of the record.
cataloguedDate	Date the record information was catalogued.
digitisedBy	Name of the person who digitised the record information into a database.
digitisedDate	Date the record information was digitised.
modifiedBy	Name of the person who last updated the record.
modifiedDate	Date of the last update to the record.
type	Type of the record, as defined by the Public Core standard.
licence	Reference to the licence under which the record is published.
institutionID	An identifier for the institution having custody of the object referred to in the record.
institutionCode	The name in use by the institution having custody of the object refrered to in the record.
collectionID	An identifier for the collection from which the record was derived.
collectionCode	The initialism identifying the collection from which the record was derived.
datasetID	An identifier for the dataset where the record is located.
datasetName	Name of the dataset where the record is located.
entryNumber	Number given to a string of records with the same sampling information in their original collection.
originalCollection	The name of the collection (person, entity or project) to which the record originally belonged.
otherCatalogNumbers	Catalogue number in the original collection.
occurrenceRemarks	Additional remarks about the record.

## Additional information

### Results

**Figure 2.**
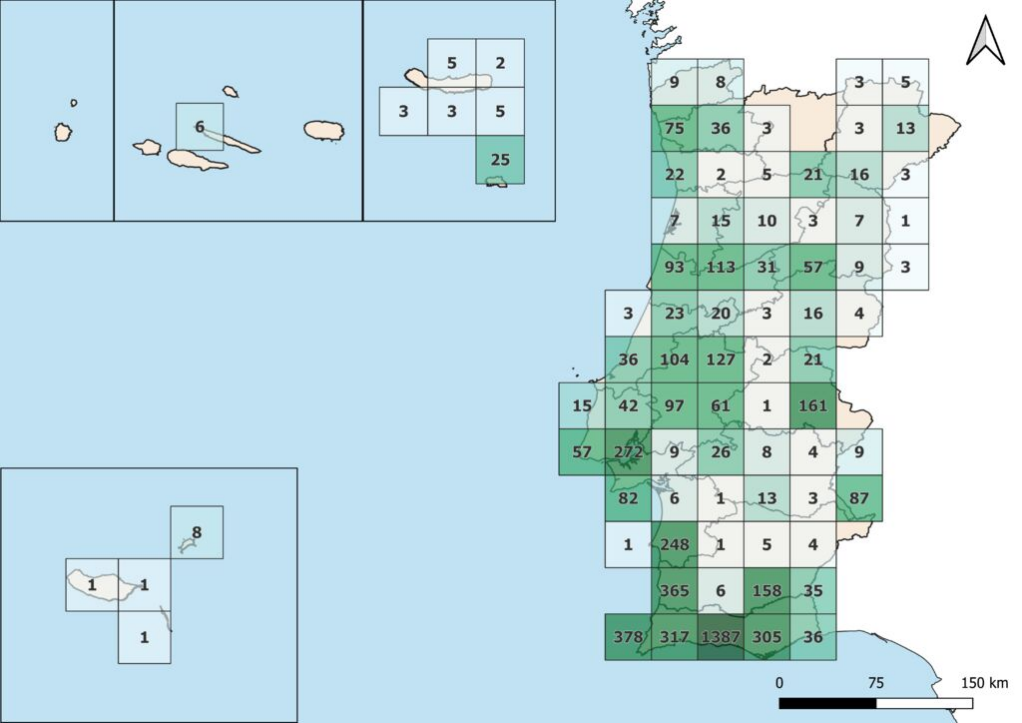
Distribution of Portuguese bee specimens in the MNHNC insect collection.

**Figure 3.**
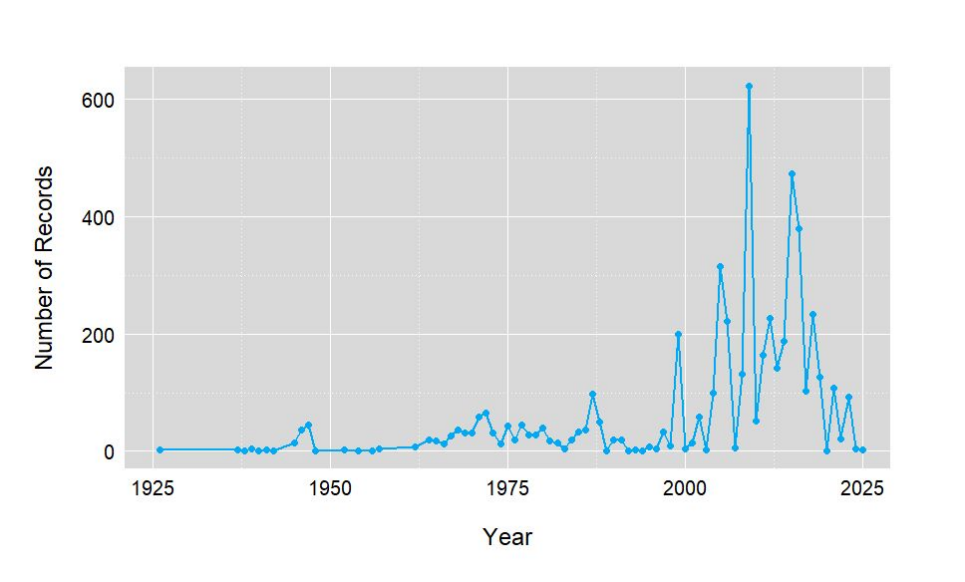
Number of records of Portuguese bees per year in the MNHNC insect collection.

The MNHNC collection holds a total of 5,295 Palearctic bee records, of which 4,830 are identified to species or subspecies level and 442 to genus or subgenus level. A substantial proportion of the Portuguese records originate from the southern and south-western coastal regions of the country (Fig. 2), with the Faro District contributing the highest number of records by a considerable margin (Table 1). The collection is largely composed of recent material, with most records collected during the 21^st^ century (Fig. 3). This pattern is partly explained by a fire that devastated the museum in 1978, destroying its zoological collections and resulting in suboptimal infrastructure for several subsequent years (Almaça 2000). Consequently, only a small number of bee records in the collection predate the 1990s (Fig. 3). In addition to Portuguese material, the MNHNC also houses 66 bee records from other Palearctic countries (Table 2).

During the cataloguing process, taxonomic identifications were revised for several genera and previously unidentified specimens were identified to species level when possible. As a result, *Nomada chrysopyga* Morawitz, 1871 — a widespread species in Central and southern Europe — is recorded here for the first time in the Portuguese territory, based on a specimen collected in Torrão, Alcácer do Sal (MNHNCENT0000872; Fig. 4). This species was recently reinstated from synonymy with *Nomada mauritanica*, a name now restricted to specimens from North Africa (Straka et al. 2024). Notably, neither *N. mauritanica* nor *N. chrysopyga* had previously been recorded in Portugal. The probable host of *N. chrysopyga*, *Andrena fuscosa* Erichson, 1835 (Smit 2018), has also been recently documented in the country (Wood et al. 2020).

Of the 54 bee genera known to occur in Portugal, 49 are represented in the Museum’s collection. The most abundant genus is *Lasioglossum* Curtis, 1833, followed by *Andrena* Fabricius, 1775 and *Eucera* Scopoli, 1770 (Table 3). According to the most recent estimate, 736 bee species were recognised from mainland Portugal (Gaspar et al. 2025); however, *Hylaeus convergens* Dathe, 2000 was subsequently removed from the national fauna following a revision of the *Hylaeus garrulus* species group (Le Divelec and Michez 2025). Furthermore, the most recent checklist of European bees introduced the recognition of *Amegilla talaris* (Pérez, 1895) and *Amegilla albigena* (Lepeletier, 1841) as separate species, with both being present in Portugal, raising the national species count by one (Ghisbain et al. 2025). With the addition of the new record reported here, the current total stands at 737 species. The collection contains specimens of 425 species, representing 57.7% of the bee species currently known from mainland Portugal.

The collection also includes several species of conservation concern, for which historical occurrence data may be valuable for future conservation planning. According to the latest IUCN European Red List of Bees, the Museum houses specimens of two Endangered species: *Nomada blepharipes* Schmiedeknecht, 1882 and *Nomada fenestrata* Lepeletier, 1841; and five species classified as Vulnerable: *Bombus muscorum* (Linnaeus, 1758), *Colletes pulchellus* Pérez, 1903, *Lasioglossum virens* (Erichson, 1835), *Nomada rhenana* Morawitz, 1872 and *Trachusa interrupta* (Fabricius, 1781) (Michez et al. 2026). In addition, the recently published Red List of Invertebrates of Portugal identified further bee species of conservation concern, including the Iberian endemic *Flavipanurgus ibericus* (Warncke, 1972), classified as Vulnerable (Boieiro et al. 2023), for which both male and female specimens are represented in the collection.

### Type material

Type material in the collection includes the holotype (MNHNCENT0062867; Fig. 5) and a male paratype (MNHNCENT0062868; Fig. 6) of the recently described Iberian endemic *Hoplitis halophila* Cross, 2023.

In addition, a specimen belonging to the INIAV insect collection — currently held in deposit at the MNHNC, where it is being preserved, catalogued and digitised — is identified as *Colletes ibericus* Noskiewicz, 1936, a junior synonym of *Colletes pulchellus* Pérez, 1903 (Ortiz-Sánchez et al. 2001) and bears a co-type label (INIAVENT000003; Fig. 7). Although this specimen was identified by the species’ author prior to its formal description (Dusmet y Alonso 1932), no Portuguese material was included in the original type series (Noskiewicz 1936), indicating that the specimen does not have type status. Moreover, the specimen does not correspond to *C. ibericus*, as it differs markedly from the lectotype designated by Ortiz-Sánchez et al. (2001) and, instead, more closely matches the diagnostic characters of *Colletes dusmeti* Noskiewicz, 1936. As a consequence of this early misidentification, the specimen was erroneously cited in Portuguese bee checklists as *C. ibericus* by Dusmet y Alonso (1932) and subsequently as *C. pulchellus* by Baldock et al. (2018), based on Dusmet’s original mention. The record of *C. ibericus* in Diniz (1959) was likely also derived from Dusmet’s publication and is, therefore, probably incorrect.

The Museum formerly housed holotypes of several Afrotropical bee species; however, these were destroyed in the fire of 1978. Consequently, the holotypes of *Anthophora atriceps* Radoszkowski, 1881, *Xylocopa mixta* Radoszkowski, 1881, *Megachile pallida* Radoszkowski, 1881, *Megachile decemsignata* Radoszkowski, 1881 and *Megachile unfasciata* Radoszkowski, 1881 — together with other hymenopteran types described by Radoszkowski from Angola (Fernandes 1959) — are presumably lost.

### Concluding remarks

The diverse bee collection of the MNHNC, which includes species of conservation concern, contributes to a more comprehensive understanding of pollinator distribution in Portugal and provides essential baseline data for assessing conservation status and providing information for future ecological research conducted in the country. By publishing the Museum’s bee collection database, we aim to make this information broadly accessible to the scientific community, thereby contributing to ongoing efforts to reduce knowledge gaps in the Portuguese bee fauna. In addition, the faunistic update, presented here, further improves current knowledge of national biodiversity.

### Author ORCIDs

Martim Baptista https://orcid.org/0009-0008-9098-626X

Paulo de Sousa https://orcid.org/0000-0002-2333-6493

Roberto A. Keller https://orcid.org/0000-0003-2751-9761

## Figures and Tables

**Figure 4a.**
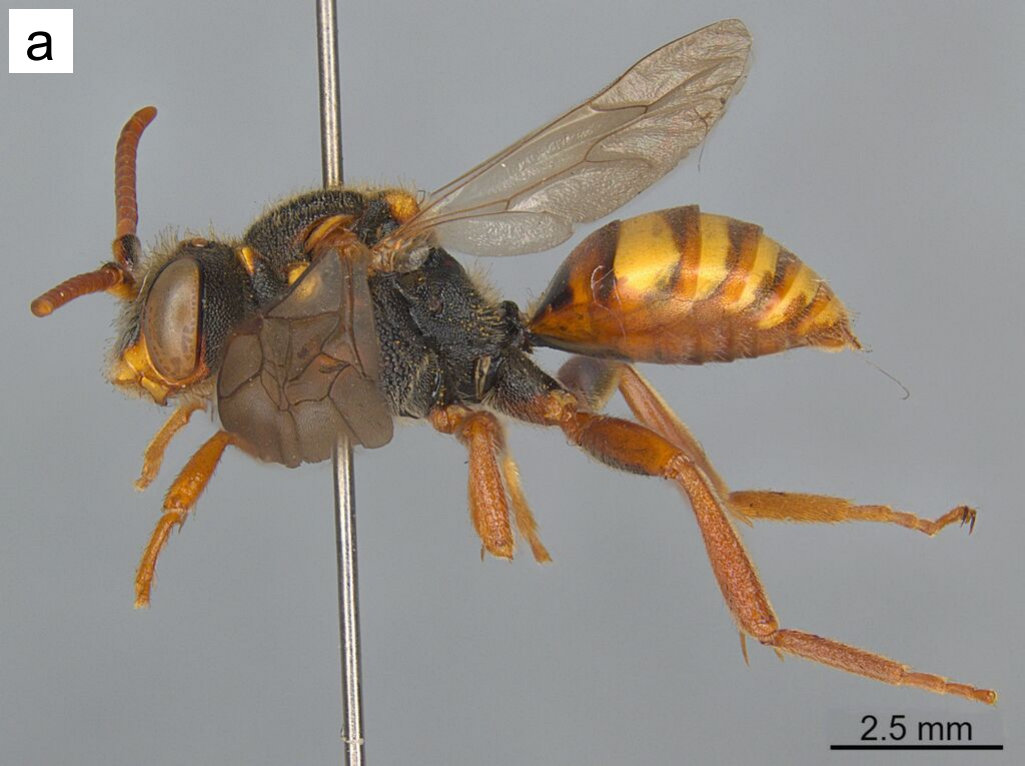
Profile View;

**Figure 4b.**
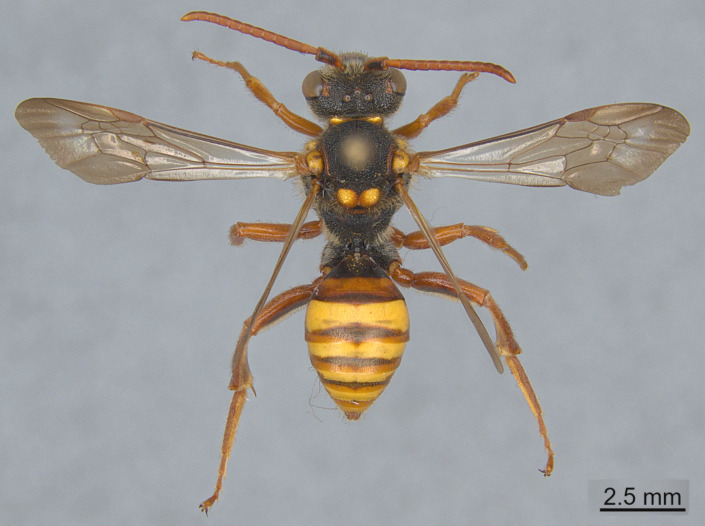
Dorsal View;

**Figure 4c.**
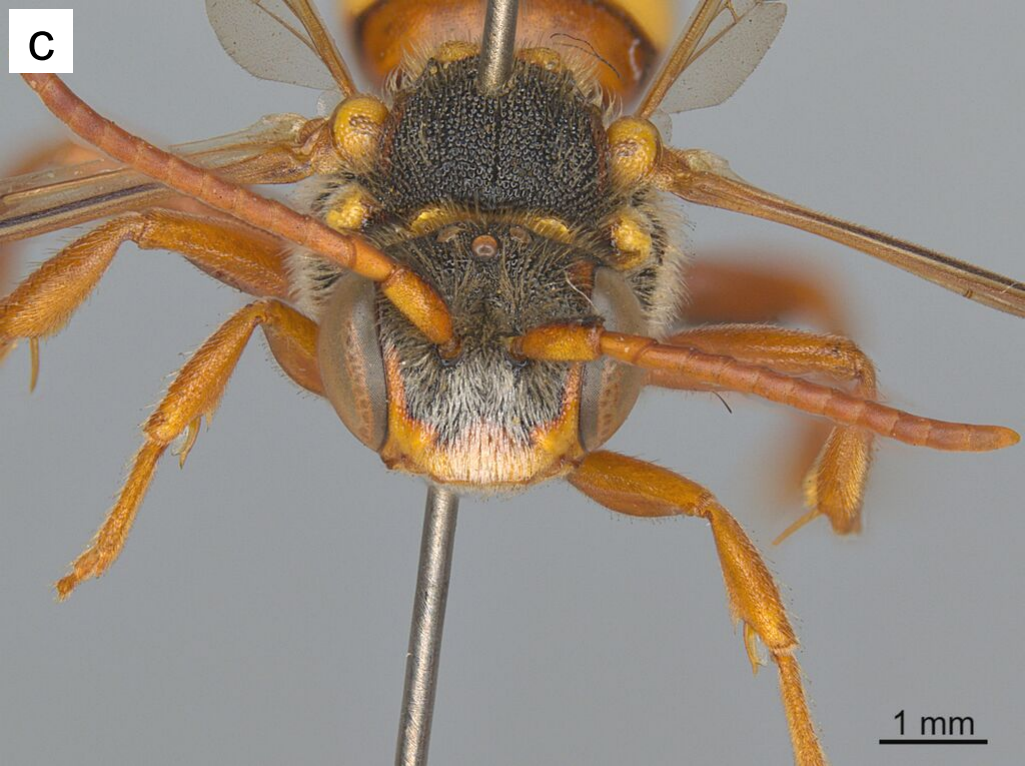
Frontal View;

**Figure 4d.**
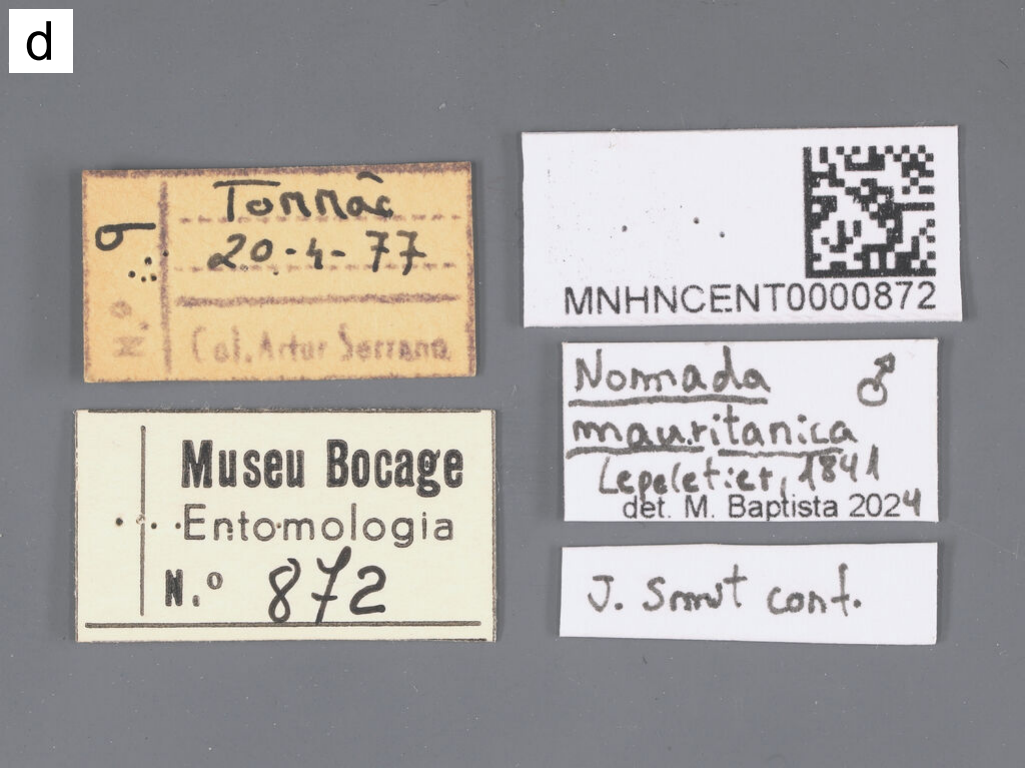
Labels.

**Figure 5a.**
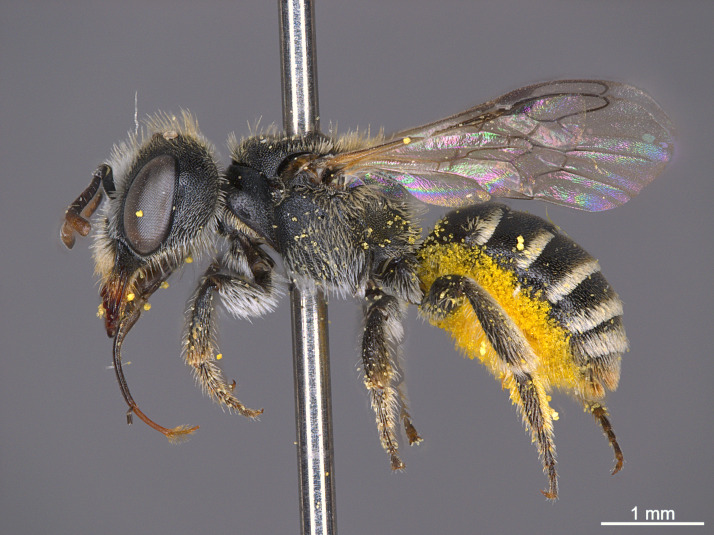
Profile View;

**Figure 5b.**
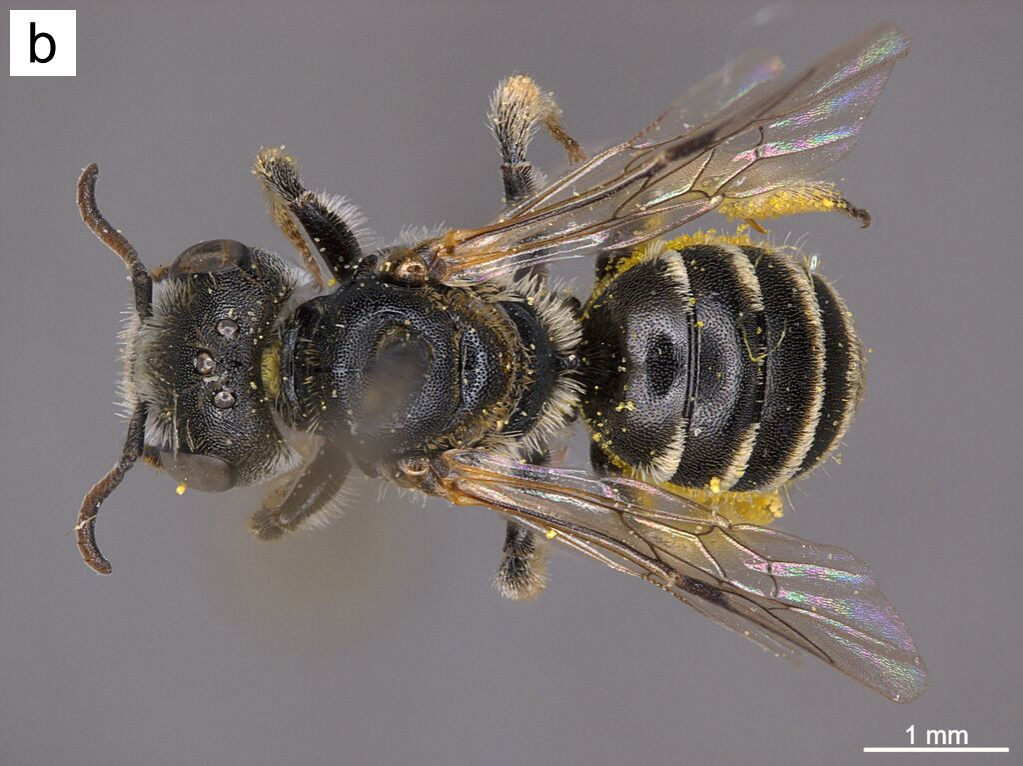
Dorsal View;

**Figure 5c.**
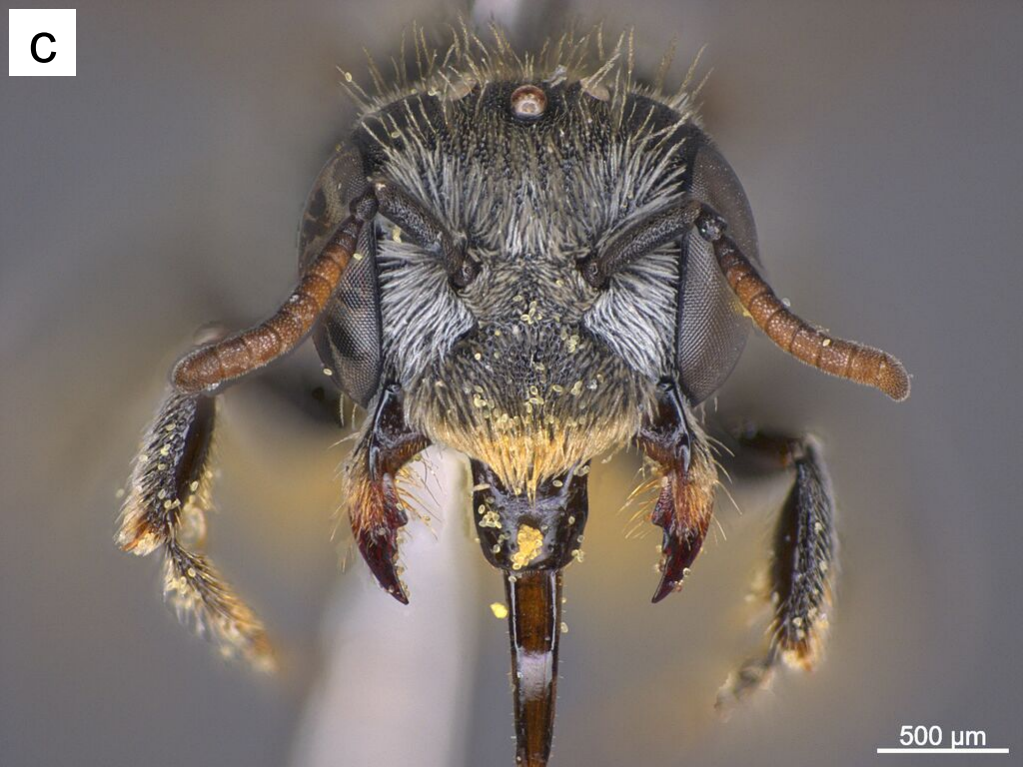
Frontal View;

**Figure 5d.**
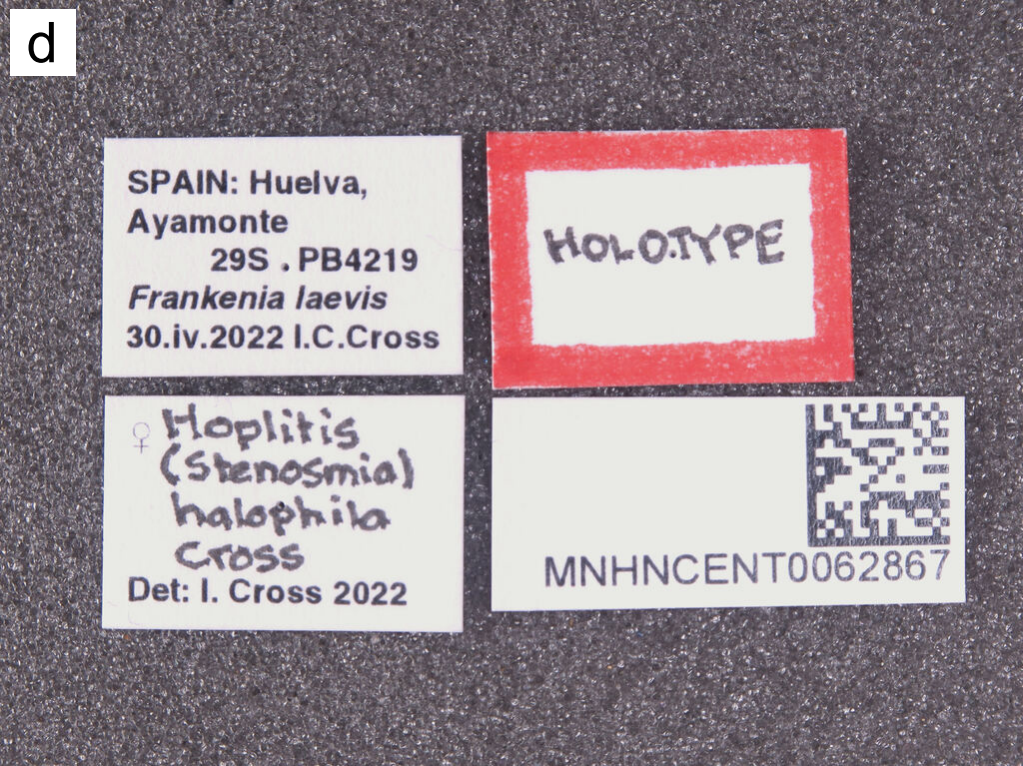
Labels.

**Figure 6a.**
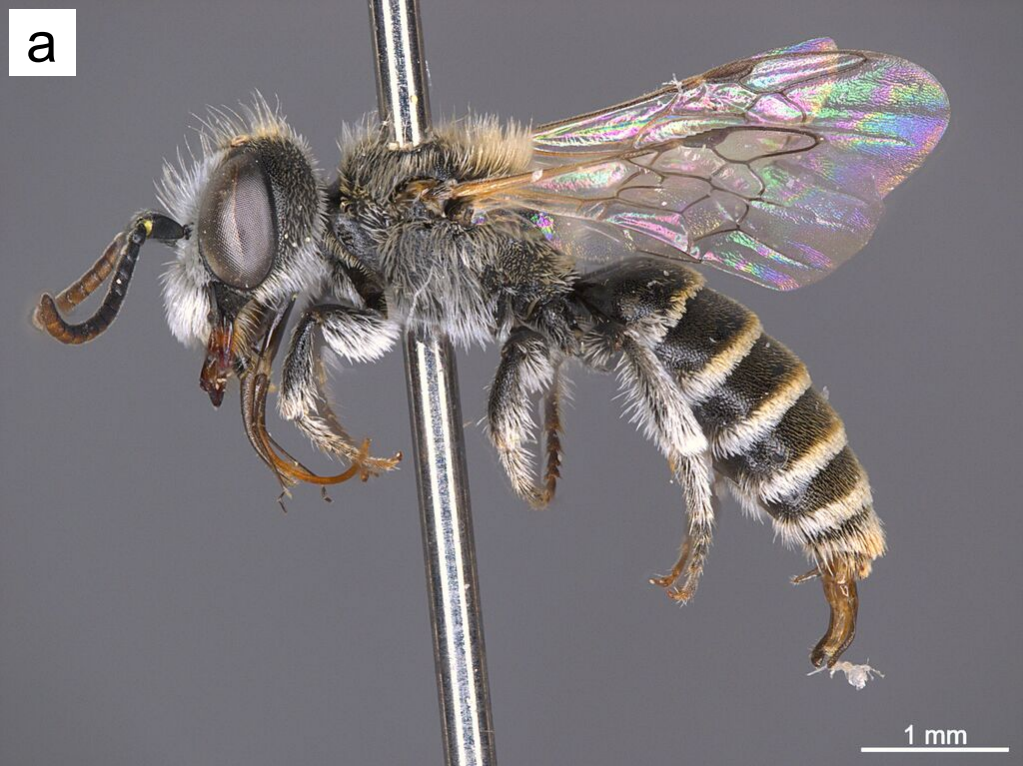
Profile View;

**Figure 6b.**
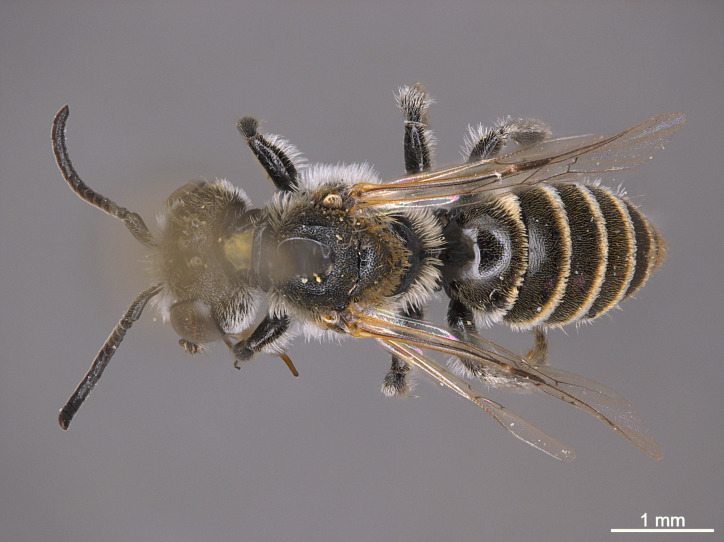
Dorsal View;

**Figure 6c.**
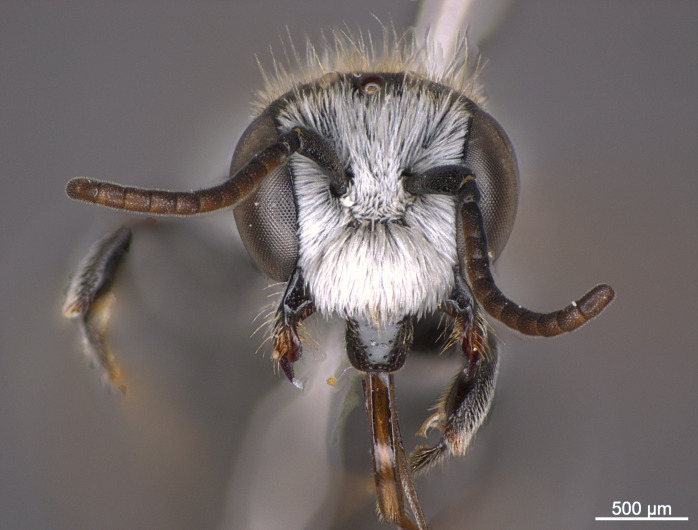
Frontal View;

**Figure 6d.**
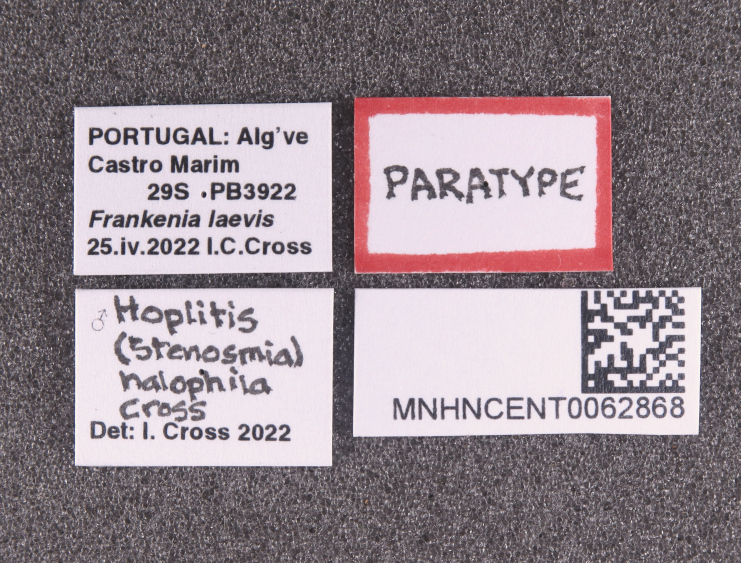
Labels.

**Figure 7a.**
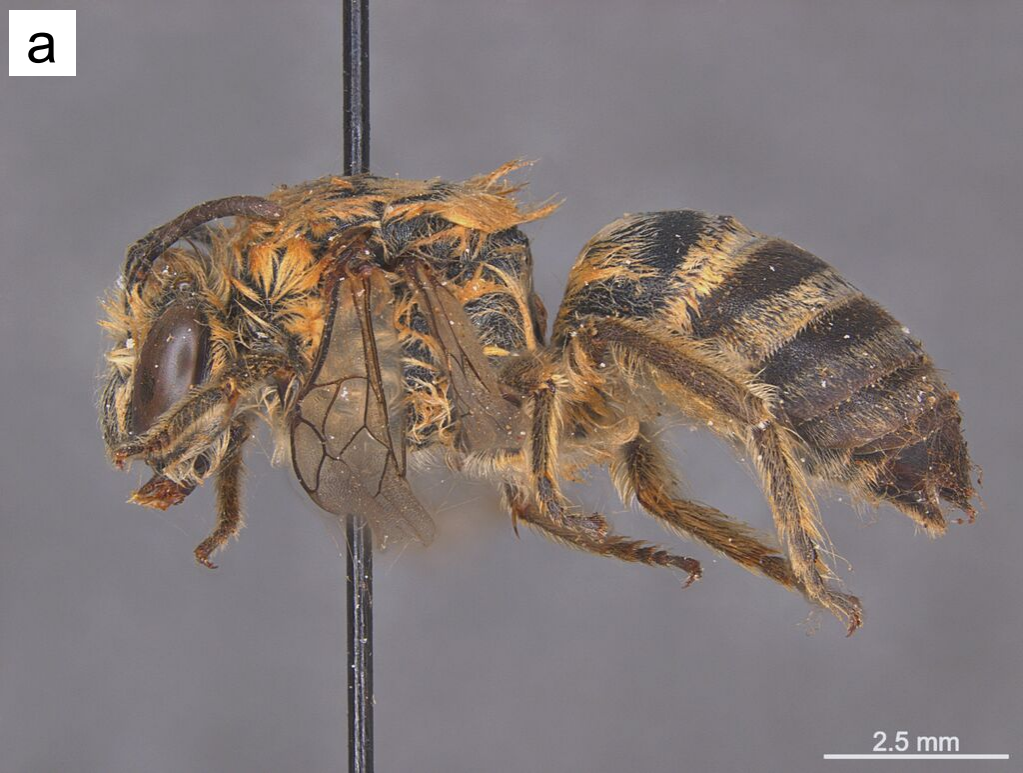
Profile View;

**Figure 7b.**
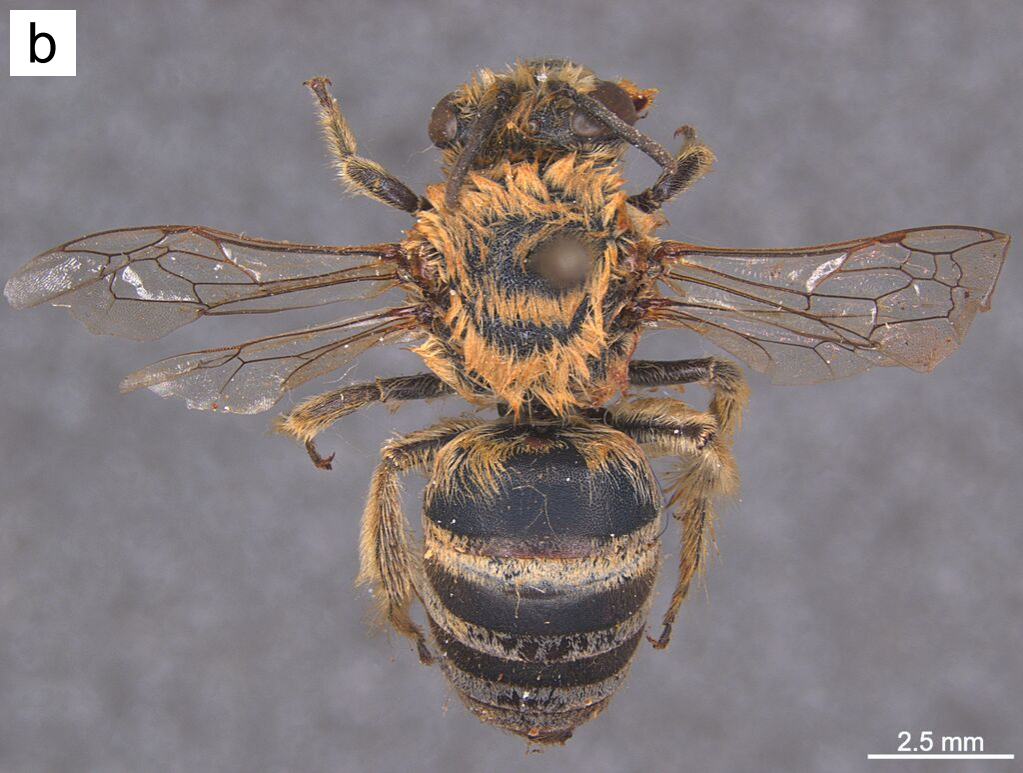
Dorsal View;

**Figure 7c.**
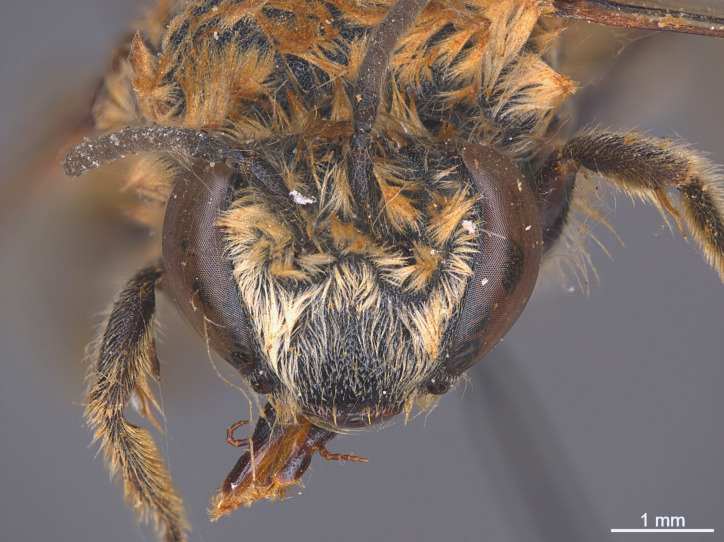
Frontal View;

**Figure 7d.**
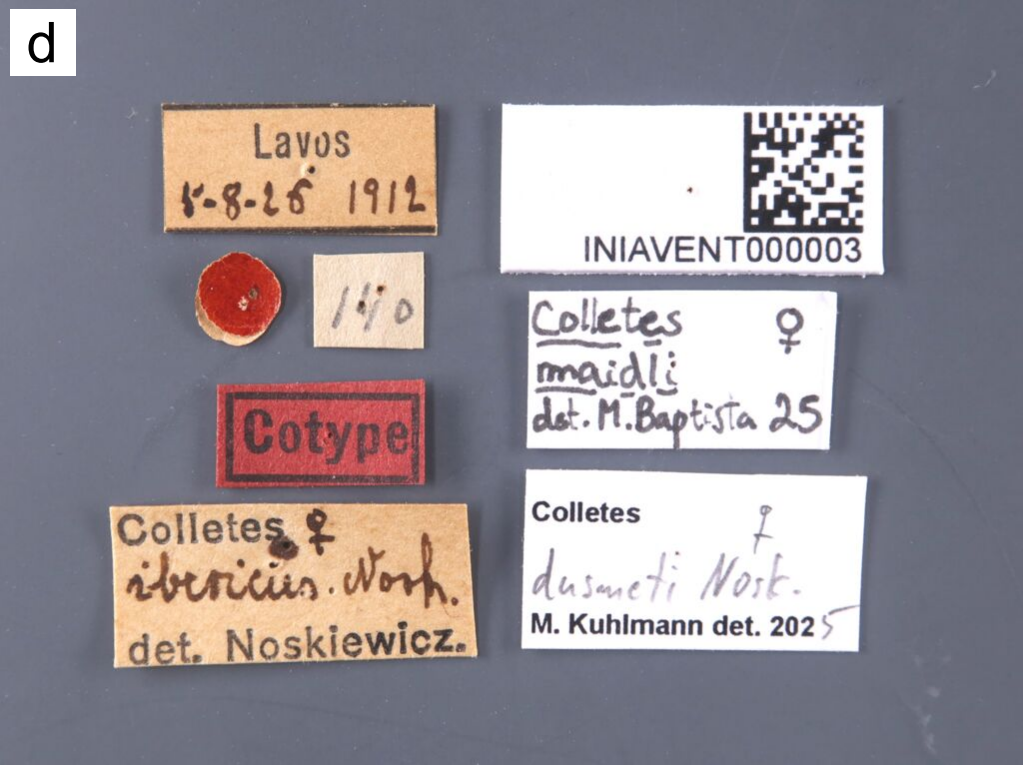
Labels.

**Table 1.** Number of bee specimens per Portuguese district in the MNHNC insect collection.

**Region**	**District**	**Number of Specimens**
Northern	Braga	95
	Bragança	43
	Porto	41
	Viana do Castelo	15
	Vila Real	3
Central	Aveiro	78
	Castelo Branco	32
	Coimbra	122
	Guarda	82
	Leiria	58
	Lisboa	301
	Viseu	119
Alentejo	Beja	529
	Évora	52
	Portalegre	224
	Santarém	393
	Setúbal	389
Algarve	Faro	2560
Autonomous Regions	Azores	49
	Madeira	27
	Unknown	7

**Table 2.** Number of bee specimens per country in the MNHNC insect collection.

**Country**	**Number of Specimens**
Portugal	5219
Spain	33
France	16
Germany	11
Belgium	4
Morocco	4
Italy	3
Hungary	1
Netherlands	1
Slovenia	1
Tunisia	1
Turkey	1

**Table 3.** Number of portuguese specimens per genus in the MNHNC insect collection.

Family	Genera	Number of Specimens
Andrenidae	Andrena	626
	Flavipanurgus	5
	Melitturga	5
	Panurginus	47
	Panurgus	272
Apidae	Amegilla	104
	Ammobates	36
	Ammobatoides	2
	Anthophora	202
	Apis	106
	Bombus	302
	Ceratina	162
	Epeolus	5
	Eucera	309
	Melecta	23
	Nomada	106
	Tetralonia	10
	Thyreus	42
	Xylocopa	130
Colletidae	Colletes	117
	Hylaeus	286
Halictidae	Ceylalictus	13
	Dufourea	13
	Halictus	223
	Lasioglossum	688
	Nomiapis	22
	Nomioides	11
	Seladonia	125
	Sphecodes	79
	Systropha	10
Megachilidae	Anthidiellum	86
	Anthidium	105
	Chelostoma	10
	Coelioxys	13
	Dioxys	10
	Heriades	93
	Hoplitis	122
	Icteranthidium	6
	Lithurgus	1
	Megachile	217
	Osmia	229
	Protosmia	6
	Pseudoanthidium	11
	Rhodanthidium	100
	Stelis	7
	Trachusa	3
Melittidae	Dasypoda	92
	Macropis	1
